# Mitochondrial dysfunctions in leukoencephalopathy with brainstem and spinal cord involvement and lactate elevation (LBSL)

**DOI:** 10.1371/journal.pone.0224173

**Published:** 2019-10-31

**Authors:** Tsu-Kung Lin, Yung-Yee Chang, Hung-Yu Lin, Chia-Wei Liou, Pei-Wen Wang, Jiin-Haur Chuang, Shang-Der Chen, Yao-Chung Chuang, Sheng-Teng Huang, Te-Yao Hsu, Cheng-Huei Peng, Min-Yu Lan

**Affiliations:** 1 Department of Neurology, Kaohsiung Chang Gung Memorial Hospital and Chang Gung University College of Medicine, Kaohsiung, Taiwan; 2 Center for Parkinson’s Disease, Kaohsiung Chang Gung Memorial Hospital and Chang Gung University College of Medicine, Kaohsiung, Taiwan; 3 Department of Pediatric Surgery, Kaohsiung Chang Gung Memorial Hospital and Chang Gung University College of Medicine, Kaohsiung, Taiwan; 4 Department of Internal Medicine, Kaohsiung Chang Gung Memorial Hospital and Chang Gung University College of Medicine, Kaohsiung, Taiwan; 5 Department of Chinese Medicine, China Medical University Hospital, Taichung, Taiwan; 6 Department of Obstetrics and Gynecology, Kaohsiung Chang Gung Memorial Hospital and Chang Gung University College of Medicine, Kaohsiung, Taiwan; University College London, UNITED KINGDOM

## Abstract

Several inherited human diseases have been linked to mitochondrial aminoacyl-tRNA synthetases (mtARSs). Leukoencephalopathy with brainstem and spinal cord involvement and lactate elevation (LBSL) is a leukodystrophy caused by mutations in the *DARS2* gene which encodes mitochondrial aspartyl-tRNA synthetase. As mitochondrial ARSs are key components of the mitochondrial translation apparatus, we investigated the effects of *DARS2* mutations on mitochondrial functions and mitochondrial morphology in an LBSL patient. In fibroblasts from the patient with LBSL, biosynthesis of respiratory chain complex proteins encoded by mitochondrial DNA was decreased, while those encoded by nuclear DNA were not. Cellular oxygen consumption rates and respiratory control ratio were decreased in the LBSL patient; in addition, fragmentation of mitochondria was increased, while their tubular elongation and interconnectivity were decreased. Taken together, these findings suggest that *DARS2* mutations impair translations of mitochondrial DNA-encoded respiratory chain complex proteins, consequently causing dysfunction of cellular respiration and impediment of mitochondrial dynamics, which highlights the role of mtARSs in the maintenance of normal mitochondrial bioenergetics and dynamics.

## Introduction

Mitochondria are vital cellular organelles for energy production, as well as the regulation of diverse cellular processes, including heme and steroid synthesis, calcium homeostasis, redox signaling, and apoptosis [1]. Maintenance of these complex physiological functions requires integration of a wide array of mitochondrial proteins. Despite the multitude of proteins involved in proper mitochondrial functioning, mitochondrial DNA (mtDNA) encodes only 13 protein subunits of respiratory chain (RC) complexes, and transfer RNA (tRNA) and ribosomal RNA for mtDNA-specific translation. Hundreds of additional gene products, including RC complex components and those necessary for mtDNA replication, translation, and maintenance are nuclear-coded [2]. Aminoacyl-tRNA synthetases (ARSs) charge amino acids to their cognate tRNA molecules in the cytoplasm and mitochondria for initiation of protein translation [3]. In humans, mitochondrial ARSs (mtARSs) are all encoded by the nuclear genome, translated in cytoplasm, and then imported into mitochondria. These mtARSs are key components of the mitochondrial translation apparatus and crucial for the expression of mitochondrial genes.

A growing number of human diseases linked to mtARSs, which predominantly affect the nervous system, have been reported in recent years [4]. Leukoencephalopathy with brainstem and spinal cord involvement and lactate elevation (LBSL) is the first disease recognized to have been associated with an mtARS gene. It is an autosomal recessive leukodystrophy caused by mutations in the *DARS2* gene, which encodes mitochondrial aspartyl-tRNA synthetase [5]. Clinically, LBSL presents early-onset progressive pyramidal, cerebellar and dorsal column dysfunction, and variable developmental delay, cognitive impairment, epilepsy, and peripheral neuropathy, without extraneural systemic manifestations [6–7]. The white matter lesions are characterized by lactate elevation and selective interference to the nerve tracts in the brainstem and spinal cord [6].

Research has demonstrated that mitochondrial dysfunction is implicated in the failure of oligodendrogenesis, and propagation of demyelination [8–11]. In the present study, we investigated the effects of *DARS2* mutations on the mitochondrial functions involved in the biosynthesis of RC complex components and cellular respiratory function in LBSL. Since dysfunction of mitochondrial dynamics, including mitochondrial fusion and fission, have been demonstrated in several inherited leukodystrophies [12–14], we also assessed mitochondrial morphology associated with the disease. Myoclonic epilepsy with ragged-red fibers (MERRF), a multi-systemic mitochondrial disease in which we previously demonstrated abnormalities of mitochondrial bioenergetics and morphology [15], was used as a positive control for mitochondrial dysfunction.

## Materials and methods

### Patients

We have previously reported on an LBSL patient who presented progressive spastic paraparesis from her teenage years, and the typical pattern of white matter changes identified by brain MRI [16]. Genetic study identified compound heterozygous mutations in the *DARS2* gene, including a splicing site mutation (c.228–16 C>A) which skips transcription of exon 3 in one allele and loss of exon 12 in the other. A patient with MERRF, caused by the m.8344A>G mutation in the mitochondrial lysyl-tRNA gene, was included in the study for comparison. This study was approved by the Ethics Committee and Institutional Review Board of Chang Gung Memorial Hospital (103–6985A3).

### Genetic study

Mutations in the *DARS2* gene and mRNA transcripts were probed with Sanger sequencing. A TaqMan^®^ copy number variation assay (Applied Biosystems, Waltham, MA, USA) was used to confirm copy number of *DARS2* exon 12 [16].

### Fibroblast culture

Skin fibroblasts from the patients with LBSL and MERRF were obtained according to the Helsinki Declarations of 1964, as revised in 2001. Normal fibroblasts derived from newborn foreskins were purchased from Millipore (EMD Millipore Corporation, CA, USA). Fibroblasts were cultured at 37 °C in DMEM (4.5 g/L, Gibco, Carlsbad, CA, USA), supplemented with 10% fetal bovine serum (Gibco), GlutaMAX (Gibco) and Antibiotic-Antimycotic (Gibco).

### Western blotting

Cells were lysed in RIPA lysis buffer (50 mM Tris, pH 7.4; 150 mM NaCl; 1 mM PMSF (phenylmethanesulfonylfluoride); 1 mM EDTA (ethylenediaminetetraacetic acid); 1% Triton X-100; 1% sodium deoxycholate; 0.1% SDS) with the addition of Protease Inhibitor Cocktail (Roche Diagnostics, Penzberg, Germany) and Phosphatase Inhibitor Cocktail I (Sigma, St. Louis, MO, USA). The antibodies used included anti-NDUFA9 (Abcam plc, Cambridge, UK), anti-COXIV (Abcam plc, Cambridge, UK), anti-COXII (Abcam plc, Cambridge, UK), anti-ND5 (GenTex, San Antonio, TX), anti-GAPDH (GenTex, San Antonio, TX), and peroxidase-labeled anti-rabbit IgG (H + L) secondary antibody (Abcam plc, Cambridge, UK). The signals were developed by ECL plus (GE Healthcare Bio-Sciences AB, Uppsala, Sweden) using X-ray films. The signal intensity was analyzed by Image J software. Respective densitometries of NDUFA9, COX IV, ND5, and COX II were quantified and normalized to the level of GAPDH using Image J.

### Oxygen consumption rate

Analysis of the oxygen consumption rate (OCR) has previously been described [17]. Briefly, OCR was monitored with a Clark electrode (Mitocell S200 micro respirometry system; Strathkelvin Instruments, Motherwell, UK). Cells (100 μL at 5×106 cells/ml) in KCl medium (100 mM KCl, 3 mM MgCl2, 20 mM, HEPES, 1 mM EDTA, 5 mM KH2PO4, pH7.4) were permeabilized by digitonin (optimal concentration of 32.5μg/ml determined by trypan blue staining) and loaded into a 200 μL MT200 Respirometer Chamber, suspended by a fixed-speed solid-state magnetic stirrer inside the chamber, and maintained at 37°C by a circulating water bath. Basal, stimulated and maximal OCR were determined by adding glutamate/malate (10 mM), ADP (0.2 mM) and Carbonyl cyanide-4-(trifluoromethoxy) phenylhydrazone (FCCP, 1 μM). All of the above reagents were purchased from Sigma (Sigma, St. Louis, MO, USA).

### Quantitative mitochondrial morphometric analysis

To observe mitochondrial morphology, cells seeded onto 35 mm glass bottom dish were stained using 100 nM Mitotracker Green FM (Invitrogen, Carlsbad, CA, USA) for 30 min at 37°C. Images were taken under a confocal microscope (FluoView FV10i, Olympus). For quantification, at least 100 cells from three independent experiments were analyzed for their mitochondrial morphology. Cells were classified into three groups according to the content of primarily “fragmented”, “short tubular” (< 5 μm in length) or “long tubular” (≥ 5 μm in length) mitochondria. Aspect ratio, elongation, and interconnectivity of individual mitochondria were measured using the Particle Analyzer plugin for Image J, as described by Dagda et al [18]. All measurements were calculated from z-axis confocal stacks. Aspect ratio calculated by major/minor axis ratio was used to determine the roundness of mitochondria. Mitochondrial elongation was measured by inverse of circularity. Mitochondrial interconnectivity was estimated by area/perimeter ratio.

### Statistical analysis

Data collected from at least three independent experiments are expressed as the mean ± SEM. Differences between two data sets were evaluated by two tailed unpaired Student’s t-test. Statistical tests between multiple data sets were analyzed using a one-way analysis of variance (ANOVA) followed by post-hoc Bonferroni’s test. A p-value < 0.05 was considered statistically significant.

## Results

**Fig 1:**
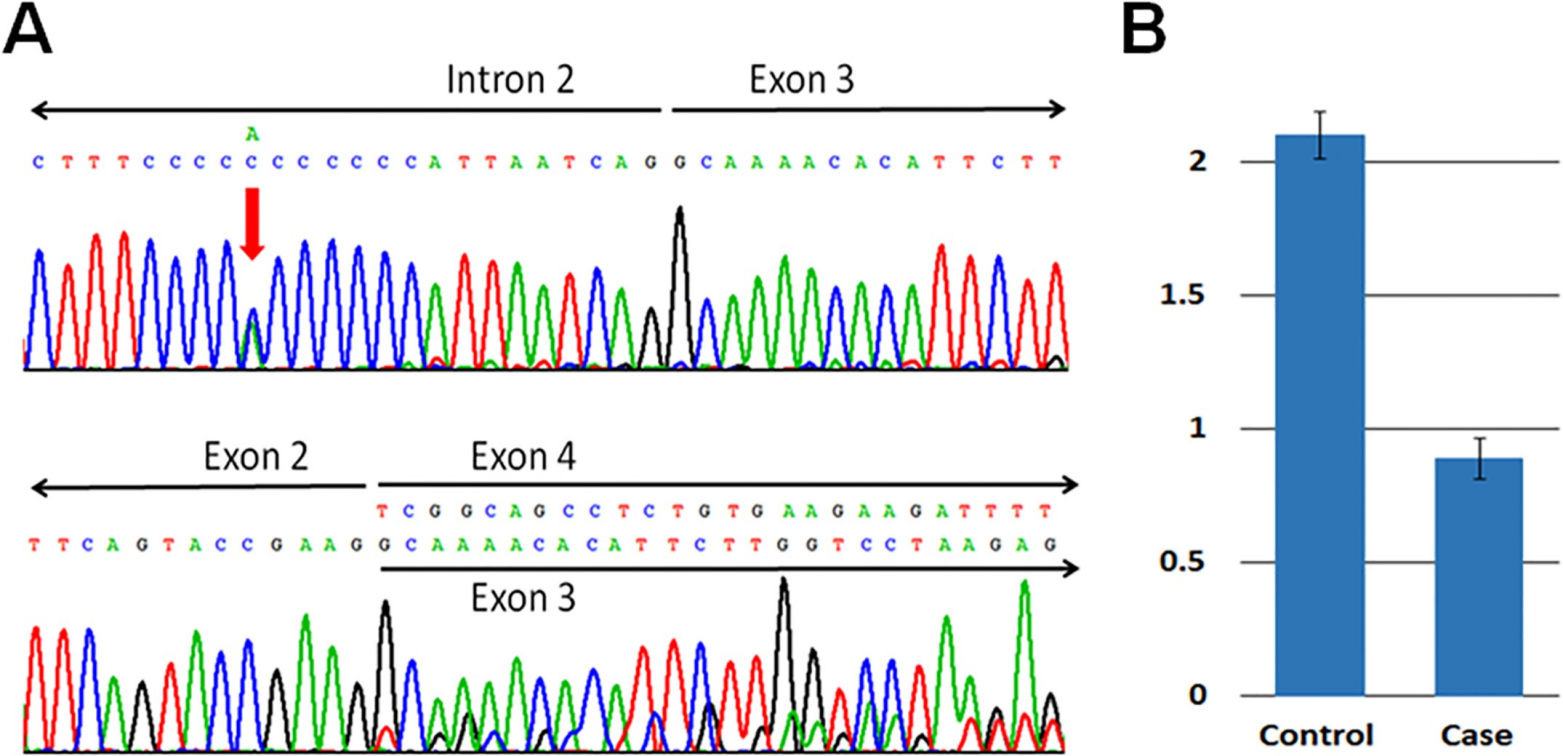
Genetic study of the LBSL patient. A *DARS2* mutation c.228–16 C>A (upper panel, indicated by arrow) in intron 2 was identified, which causing a *DARS2* mRNA transcript lacking exon 3 (lower panel); (B) TaqMan copy number assay (vertical bars indicating the ranges of values) for *DARS2* exon 12 showed loss of a copy of exon 12.

As previously mentioned, the LBSL patient harbors the c.228–16 C>A mutation in intron 2 (upper panel of Fig 1A) which results in a RNA transcript lacking exon 3 (lower panel of Fig 1A), and one copy loss of exon 12 (Fig 1B) in the *DARS2* gene. The compound heterozygous mutations are consistent with an autosomal recessive inheritance pattern for LBSL.

**Fig 2:**
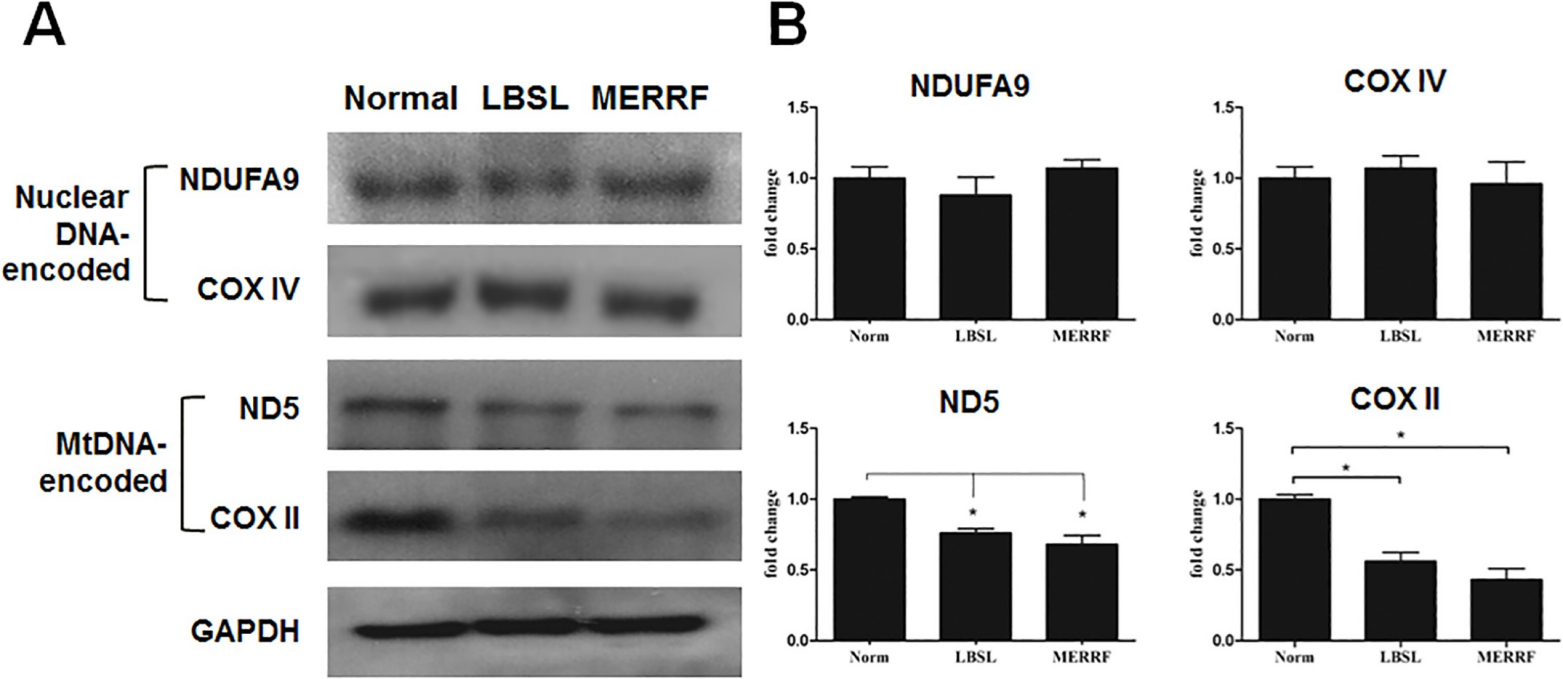
DARS2 defect leads to reduced mitochondrial translation. (A) Representative image of immunoblotting. Nuclear and mitochondria DNA-encoded proteins of mitochondrial respiratory complexes in fibroblasts derived from normal human, LBSL and MERRF patient were used for assay. (B) Quantitative histogram of protein expression. *p<0.05 between indicated groups.

In the investigations for synthesis of RC complex components, the fibroblasts of the LBSL patient had lower levels of mtDNA-encoded proteins ND5 (0.76±0.05%) and COX II (0.66±0.02%) compared to normal fibroblasts (Fig 2A and 2B). In contrast, nuclear-encoded RC complex proteins, including NDUFA9 and COX IV, did not demonstrate significant differences between the LBSL and normal fibroblasts. This disparity in synthesis of mtDNA-encoded and nuclear-encoded RC complex proteins was similar to those presented by the fibroblasts from MERRF patient, in which levels of ND5 (0.68±0.11%) and COXII (0.43±0.03%) were decreased, while NDUFA9 and COX IV levels were not significantly changed, when compared to normal controls.

**Fig 3:**
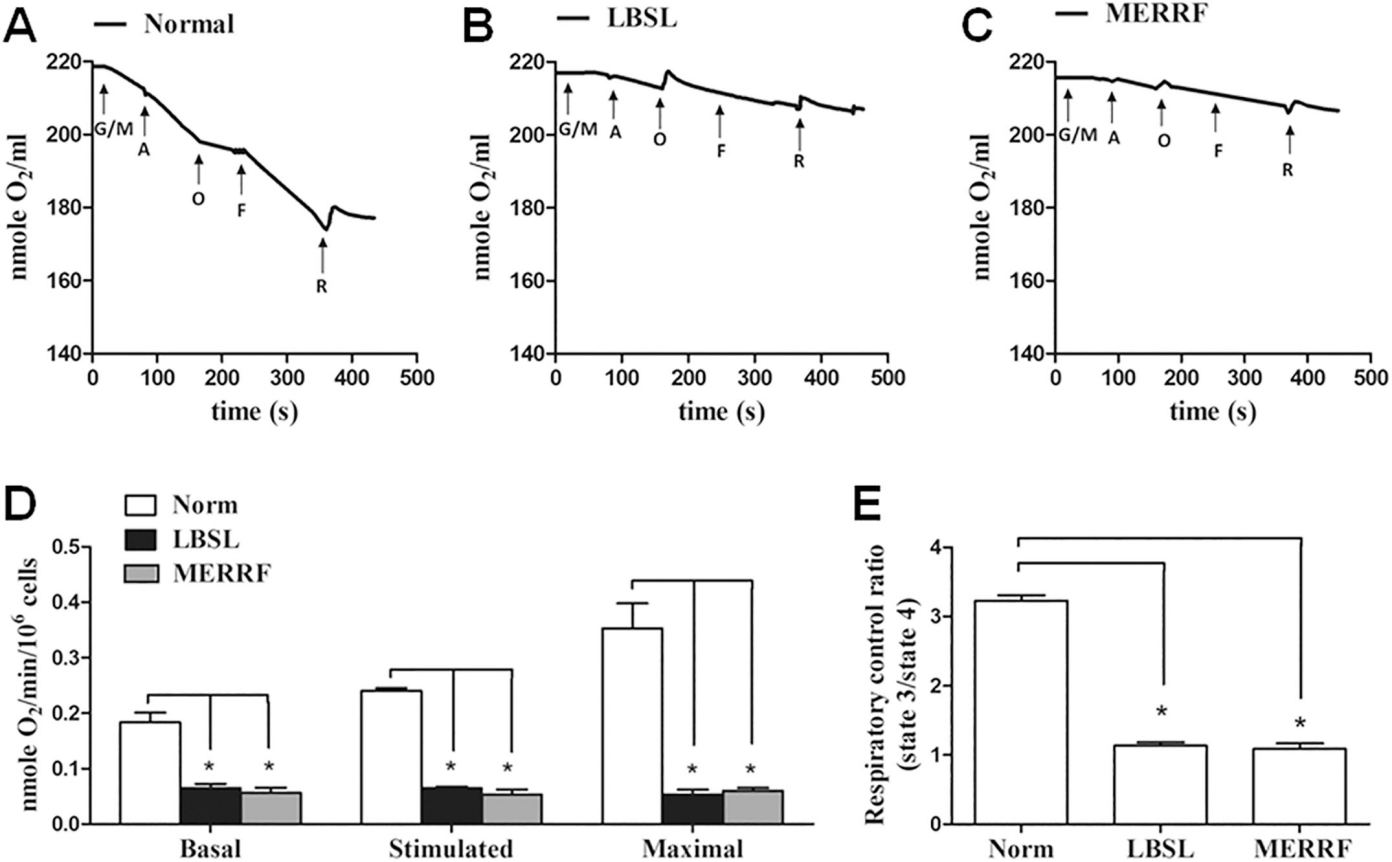
DARS2 defect causes respiratory dysfunction of mitochondria. (A, B and C) Representative results of polarographic data. Cells resuspended in oxymeter were sequentially loaded with indicated drugs to dissect respiratory capacity. G/M, glutamate and malate. A, ADP. O, oligomycin, F, FCCP. R, rotenone. (D) Basal, ADP-stimulated and FCCP-activated maximal respiration rate. (E) Respiratory control ratio calculated from ration of state 3 to state 4 respiration rate. State 3 and state 4 were defined as oxygen consumption under ADP and oligomycin, respectively. *p<0.05 between indicated groups.

In the cellular respiratory function analysis, the LBSL fibroblasts displayed significant decrease of basal OCR, about 65% less than normal fibroblasts (0.065 ± 0.013 vs. 0.183 ± 0.031 nmole O_2_/min/10^6^ cells) (Fig 3A–3D). Furthermore, in the LBSL fibroblasts as compared to control, stimulated OCR induced by ADP was 73% reduced (0.065 ± 0.005 vs. 0.24 ± 0.01 nmole O_2_/min/10^6^ cells), and maximal OCR induced by FCCP was 85% reduced (0.053 ± 0.015 vs. 0.353 ± 0.078 nmole O2/min/10^6^ cells). Consequently, the respiratory control ratio (OCRIII/OCRIV), which is used as an index for coupling of oxidative phosphorylation, was 65% decreased in the LSBL fibroblasts (1.13 ± 0.09, vs. 3.23 ± 0.14 in control) (Fig 3E). Likewise, the MERRF fibroblasts had lower basal (0.057±0.015 nmole O_2_/min/10^6^ cells), stimulated (0.053±0.015 nmole O_2_/min/10^6^ cells), and maximal OCRs (0.06±0.01 nmole O_2_/min/10^6^ cells), and respiratory control ratio (1.08±0.14) when compared to normal fibroblasts.

**Fig 4:**
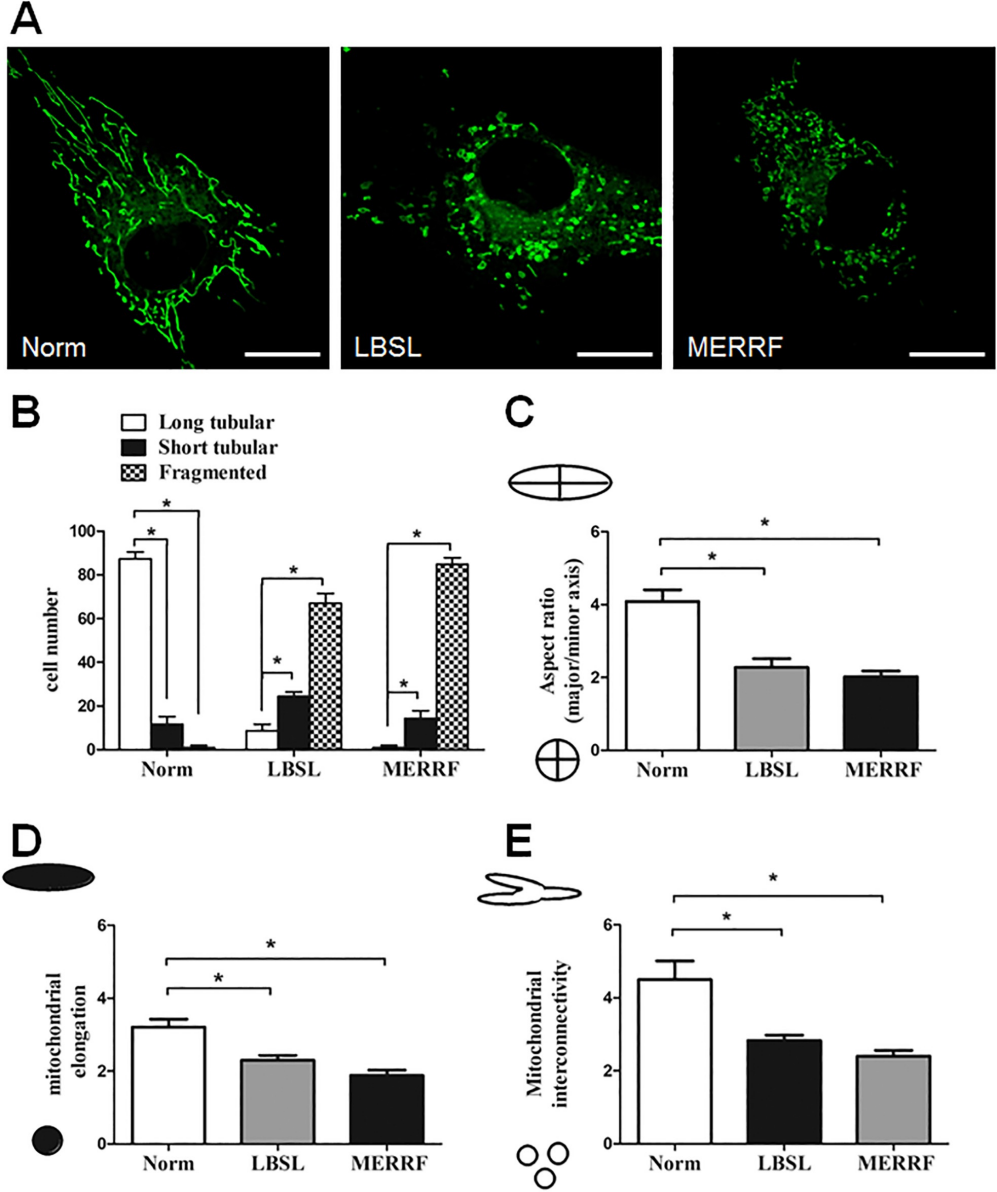
Cells with DARS2 defect present fragmented morphology of mitochondria. (A) Representative image of mitochondrial morphology. Scale bar, 10 μm. (B-E) Mitochondrial morphology was analyzed using Dagda’s method, as described in Materials and Methods. *p<0.05 between indicated groups.

We next measured the indices of mitochondrial morphology in the fibroblasts. The LBSL fibroblasts which contained fragmented and short tubular mitochondria outnumbered those with long tubular mitochondria, with 72.3% (± 4.04%) of cells containing predominantly fragmented mitochondria, 19% (± 1%) containing short tubular ones, and only 8.6% (± 3.05%) containing long tubular ones (p< 0.05). By contrast, the mitochondria are in a long tubular shape in 87% (± 3.2%) of the normal controls. Only a small portions of normal cells contained predominantly short tubular (11.6 ±3.5%) and fragmented mitochondria (1±1%) (p< 0.05) (Fig 4A and 4B). Mean aspect ratio and inverse of circularity of mitochondria in the LBSL fibroblasts were about 56% (2.277 ± 0.414 vs. 4.085 ± 0.649, p< 0.05) and 72% for control (2.297 ± 0.232 vs. 3.207 ± 0.386, p< 0.05) (Fig 4C and 4D). A measurement of area/parameter ratio suggested that the LBSL fibroblasts had 37% reduced branching mitochondrial network than control fibroblasts (2.833 ± 0.252 vs. 4.5 ± 0.889, p< 0.05) (Fig 4E). The MERRF fibroblasts were more frequently containing fragmented (84.8±3%) and short tubular mitochondria (14.3±3.5%) than containing long tubular ones (1 ±1%) (p< 0.05) (Fig 4A and 4B). Mean aspect ratio (1.89±0.03), inverse of circularity (1.87± 0.02) and area/parameter ratio (1.9±0.02) of mitochondria were decreased in the MERRF fibroblasts when compared to normal cells (Fig 4C and 4E). In summary, fragmentation was increased while tubular elongation and interconnectivity were decreased for mitochondria of the LSBL fibroblasts, which were similar to the changes of mitochondrial morphology in the MERRF fibroblasts.

## Discussion

Because both of the *DARS2* mutations harbored by the LBSL patient in this study result in loss of an entire exon in mRNA transcripts, significant alterations to the structure and function of the synthetic aspartyl-tRNA synthetase could be expected. Our research finds that cells from the LBSL patient exhibited decreased translations of mtDNA-encoded RC complex proteins ND5 and COX II. ARSs are essential components of the protein translation machinery. In addition, accuracy of aminoacylation of tRNA is an important element for translation fidelity [19]. The selectivity by which mitochondrial ARSs recognize the specific amino acid and the corresponding tRNA is an important quality control mechanism for mitochondrial protein synthesis. Thus, dysfunction of even a single mtARS may have significant impact on synthesis of multiple mtDNA-encoded RC complex subunits. Consequently, deficiencies of multiple protein subunits in the respiratory chain complexes due to *DARS2* mutations may impair oxidative phosphorylation, which is evident herein by decreases in basal, stimulated and maximal OCRs, and respiratory control ratio for ATP synthesis. Contrastingly, expressions of NDUFA9 and COX IV are normal, suggesting normal translations of nuclear-encoded mitochondrial proteins.

Under normal physiological conditions, mitochondrial fusion and fission occur in a balanced manner to remodel the mitochondrial network in response to the metabolic needs of cells. Mitochondrial fusion and fission mechanisms are crucial for quality control of mtDNA and proteins. The fusion process optimizes mitochondrial function by the spreading of molecules and mtDNA throughout the entire mitochondrial compartment [20]. Meanwhile, fission segregates damaged mitochondria and facilitates their removal by mitophagy [21]. Our data shows increased fragmentation and reduced interconnecting networks of mitochondria in cells with *DARS2* mutations, suggesting that fusion is impeded while fission is enhanced in LBSL cells. Mitochondrial morphology is often associated with the cellular energy state, as such inhibition of oxidative phosphorylation and increased oxidative stress may suppress mitochondrial fusion and induce mitochondrial fission [22–23]. Furthermore, loss of mitochondrial fusion results in a decrease of mtDNA content, loss of mitochondrial membrane potential, deficiency of oxidative phosphorylation, and reduced respiratory chain function [24–26], causing a vicious cycle that aggravates mitochondrial dysfunction in LBSL cells. Alternatively, it has been suggested that mitochondrial fission prevents the increase of heteroplasmy in cell models of mitochondrial diseases [27], therefore enhancement of mitochondrial fission could be a protective mechanism which LBSL cells apply to promote elimination of dysfunctional mitochondria through mitophagy.

Although the study demonstrates mitochondrial dysfunction in fibroblasts from the patient, clinical phenotype of LBSL does not encompass extraneural manifestations including connective tissue abnormality, probably due to lower dependence on oxidative metabolism for fibroblasts when compared to neural cells. In this study, the changes of the LBSL cells in RC complex protein translations, cellular respiratory functions and mitochondrial morphology were similar to those displayed by the MERRF cells, which were used as a positive control for mitochondrial dysfunction. However, it cannot be concluded that LBSL and MERRF share a common pathogenic mechanism in view of the marked differences in genetics (mutation of nuclear gene *vs*. mutation of mtDNA-encoded gene), inheritance mode (mendelian transmission *vs*. maternal inheritance with heteroplasmy), aminoacylation system (asparate *vs*. lysine) and clinical phenotypes (predilection of nervous system involvement *vs*. multi-systemic manifestations) of the two diseases. Furthermore, it remains unclear why *DARS2* mutations clinically cause the selective involvement of central nervous system white matter. Intriguingly, apart from LBSL, several other mtARS gene mutations have been linked to the development of leukodystrophy [4]. For different cell types harboring the most common *DARS2* mutation, the splicing site mutation 228–20_-21delTTinsC in intron 2, skipping of exon 3 transcription is more pronounced in neuronal cells than in oligodendrocytes [28]. In a transgenic mice model, loss of DARS2 expression leads to more severe apoptosis in neuronal cells compared to myelin-producing cells, although respiratory chain deficiency is similar in the two types of cells [29]. These findings suggest that LBSL might originate from the primary neuronal and axonal defects due to *DARS2* mutations, with oligodendrocyte dysfunction and demyelination being secondary effects. An alternative explanation might be that expression of mitochondrial genes is crucial for oligodendroglial differentiation [30], and its dysfunction caused by *DARS2* mutations may repress oligodendrocyte-mediated myelination in the developing brain. This hypothesis could be plausible in view of neonatal onset of the disease in some LBSL cases [31].

This study demonstrates that *DARS2* mutations in LBSL cells impair translation of mtDNA-encoded RC complex proteins, which in turn causes dysfunction of cellular respiration and impediment of mitochondrial dynamics. These findings highlight the role of mtARSs in the maintenance of normal mitochondrial bioenergetics and dynamics. The functional implications of mitochondrial ARSs in myelin synthesis and maintenance of the central nervous system are as yet unknown, and merit attention in future investigations of the pathogenesis of LBSL and other mitochondrial ARSs-associated leukodystrophies.

## Supporting information

S1 Data Western blot, in “Minimal data set_Western.sav”. (SAV)

S2 Data Cell respiratory functions, in “Minimal data set_Resp.sav”. (SAV)

S3 Data Mitochondrial morphology, in “Minimal data set_Morph.sav”. (SAV)
